# Evolution of Biologically Related Living Kidney Donation in the United States from 1988 to 2022

**DOI:** 10.1681/ASN.0000000000000424

**Published:** 2024-05-29

**Authors:** Fawaz Al Ammary, Simeon Adeyemo, Krista L. Lentine, Abimereki D. Muzaale

**Affiliations:** 1 Department of Medicine, University of California Irvine, Orange, California; 2 Department of Medicine, Saint Louis University, St. Louis, Missouri; 3 Department of Surgery, Johns Hopkins University, Baltimore, Maryland

**Keywords:** kidney donation, kidney transplantation, minority health and disparities, nephrectomy

## Introduction

Nearly 100,000 patients in the United States await kidney transplantation. Living donation reduces the wait and offers superior patient and graft survival compared with deceased donation. Yet, living kidney donors have declined over the past 2 decades, mainly driven by declining biologically related donors with the recipient.^1^ Evolving knowledge of postdonation long‐term risks, including concern for familial risk of CKD and kidney failure, may explain, in part, the declining trends since 2005, along with a complex interplay of nonclinical factors.^2–4^ However, this decline remains unsupported and needs further exploration. We sought to understand the interplay of biological donor–recipient relationship and race to guide future interventions reversing these trends and increase equity in access to living donor kidney transplantation.^5^ We studied 106,033 biologically related living kidney donors from 1988 to 2022, by race/ethnicity.

## Methods

**Figure 1:**
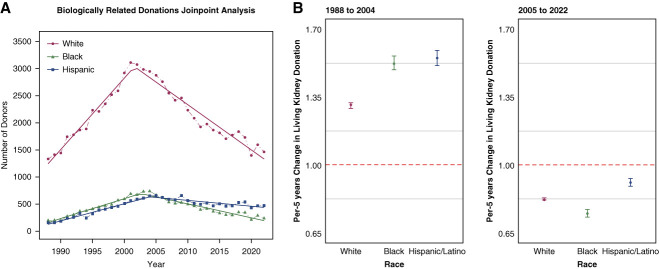
**The change in biologically related living kidney donations in the United States from 1988 to 2022.** (A) Trends in biologically related living kidney donations in the United States from 1988 to 2022, using joinpoint trend analysis. The dotted lines represent the observed data, and the straight lines represent the fitted model of the data. (B) IRR of biologically related living kidney donation, stratified by era. Per 5-year change in number of biologically related living kidney donors estimated from Poisson regression model. An IRR greater than one indicates an incline each year in the number of living kidney donors. The number of biologically related donors increased between 1988 and 2004 and decreased between 2005 and 2022 across race/ethnicity. The category of White included 93% Caucasian, 5% Asian, and 2% others including American Indian or Alaskan Native, Native Hawaiian, or other Pacific Islander, and multiracial. All analyses were performed using R statistical software (v4.2.2; R Core Team 2022). IRR, incident rate ratio.

We conducted a retrospective cohort study using the US national registry of donors (Scientific Registry of Transplant Recipients [SRTR]). The SRTR data system includes data on all donors, waitlisted candidates, and transplant recipients in the United States. The outcome of interest was the change in biologically related living kidney donations over time. *A priori*, we stratified the analyses by era (1988–2004 and 2005–2022) and donor race as self-reported (Black, Hispanic, and White/others). According to SRTR data, living kidney donors began to decline in 2005, hence our choice to dichotomize into two eras before and after 2005 supported by joinpoint analysis (Figure 1A). We used Poisson regression to estimate the change in number of biologically related donors per 5-year increment (incidence rate ratio [IRR]). We excluded 25 donors from our study because of missing data on race/ethnicity (0.024%).

## Results

Of 106,033 biologically related donors (43% full sibling, 25% offspring, 20% parents, and 12% non–first-degree relative donors), 71% were White, 14% Black, and 15% Hispanic. The median age was 39 years, interquartile range 30–47, and 58% were female. From 1988 to 2004, the absolute number of White related donors increased 2.2-fold (1332 to 2954), Black related donors increased 3.8-fold (197–740), and Hispanic related donors increased 4.2-fold (155–647). Conversely, from 2005 to 2022, White related donors decreased 49% (2884 to 1463), Black related donors decreased 64% (667–238), and Hispanic related donors decreased 28% (654–470). The proportion of biologically related donors decreased from 79% in the period between 1988 and 2004 to 49% in the period from 2005 to 2022.

The rates of biologically related donors between 1988 and 2004 increased across race/ethnicity. For every 5-year increment, White related donors increased by 31% (IRR _1.29_1.31_1.32_), Black related donors increased by 52% (IRR _1.49_1.52_1.56_), and Hispanic related donors increased by 55% (IRR _1.51_1.55_1.59_). Conversely, the rates of biologically related donors between 2005 and 2022 decreased across race/ethnicity. For every 5-year increment, White related donors decreased by 18% (IRR _0.82_0.82_0.83_), Black related donors decreased by 25% (IRR _0.73_0.75_0.77_), and Hispanic related donors decreased by 9% (IRR _0.89_0.91_0.93_) (Figure 1B).

In a sensitivity analysis, these inferences remained unchanged when we excluded biologically related living kidney donations for pediatric (<18 years) recipients. The concern was that pediatric transplant candidates had received priority for high‐quality deceased donor kidneys since 2005, potentially explaining the decline in biologically related donors, which was not the case.

## Discussion

In this national study of biologically related living kidney donors, we found a steady increase from 1988 to 2004, followed by a sustained decline from 2005 to 2022 across race/ethnicity. White donors account for most of this decline. Previous studies show that the number of unrelated White donors has increased, partially offsetting this trend. However, this compensation phenomenon is not true for Black and Hispanic donors.^1,6^ The net effect of these trends is a lack of US living kidney donations.

Our findings of persistent declining biologically related donors over the past 20 years call for efforts to understand modifiable barriers. Lack of education can create misconceptions about postdonation risks. Socioeconomic factors might also have played a role,^7^ knowing the Great Recession hit in the late 2000s. Kidney failure is more prevalent in individuals with low socioeconomic status, and potential donors from their families may face challenges in donating a kidney. Financial barriers to living donation have been a long-standing problem. Efforts are needed to reduce donor travel costs, lost wages, and dependent care expenses. Telemedicine services can make donor evaluation and follow-up care more accessible and efficient. Furthermore, the evolution in donor–recipient relationship patterns illustrates the need to eliminate recipient means testing as a criterion for National Living Donor Assistance Center eligibility. Moreover, biologically related donors may share risk factors, for example, diabetes, hypertension, obesity, or genetic risk, which could disqualify them as donors. Nonetheless, a family history of kidney disease in itself is not a contraindication to donation, but related donor candidates may warrant a more detailed assessment depending on the recipient's kidney disease.

Our study has limitations. The data available cannot explain the causes contributing to the sustained decline in donations starting from 2005. Furthermore, stratifying the analyses by era, although justifiable, may introduce period dichotomization bias because this assumes uniform external influences within each era, potentially oversimplifying complex trends. Mandated US transplant registry data do not capture information on donors who were evaluated but deemed ineligible, thus preventing assessment of donor exclusions on the basis of factors including familial risk. The SRTR Living Donor Collective candidate registry would be helpful in that regard.

This study highlights the opportunity for programs to invest in reducing barriers to biologically related donations, especially for healthy adults in racial/ethnic minorities. Our findings highlight alarms for clinical practice and policy makers to reduce hurdles biologically related donors face, potentially by providing education, easing access to donor evaluation, offering social and financial support, and assuring postdonation follow-up care.

## Data Availability

Partial restrictions to the data and/or materials apply. Data are available from the Scientific Registry of Transplant Recipients (SRTR) with authorized permission.
